# Young Woman with a Fever and Chest Pain

**DOI:** 10.5811/westjem.2016.1.29233

**Published:** 2016-03-02

**Authors:** Kristin H. Dwyer, Joshua S. Rempell

**Affiliations:** Brigham and Women’s Hospital, Department of Emergency Medicine, Boston, Massachusetts

A 26-year-old female presented to the emergency department with three days of subjective fevers, dry cough and pleuritic chest discomfort. On exam, her vital signs were significant for a heart rate of 106/minute and oxygen saturation of 95% on room air. Her lung exam revealed decreased breath sounds at the right base. A bedside lung ultrasound and a chest radiograph were performed (Figure 1a, Figure 2, and Video).

## DIAGNOSIS: COMMUNITY ACQUIRED PNEUMONIA

Community acquired pneumonia (CAP) is a common disease in the United States and represents the seventh leading cause of death.^1^

**Figure 2:**
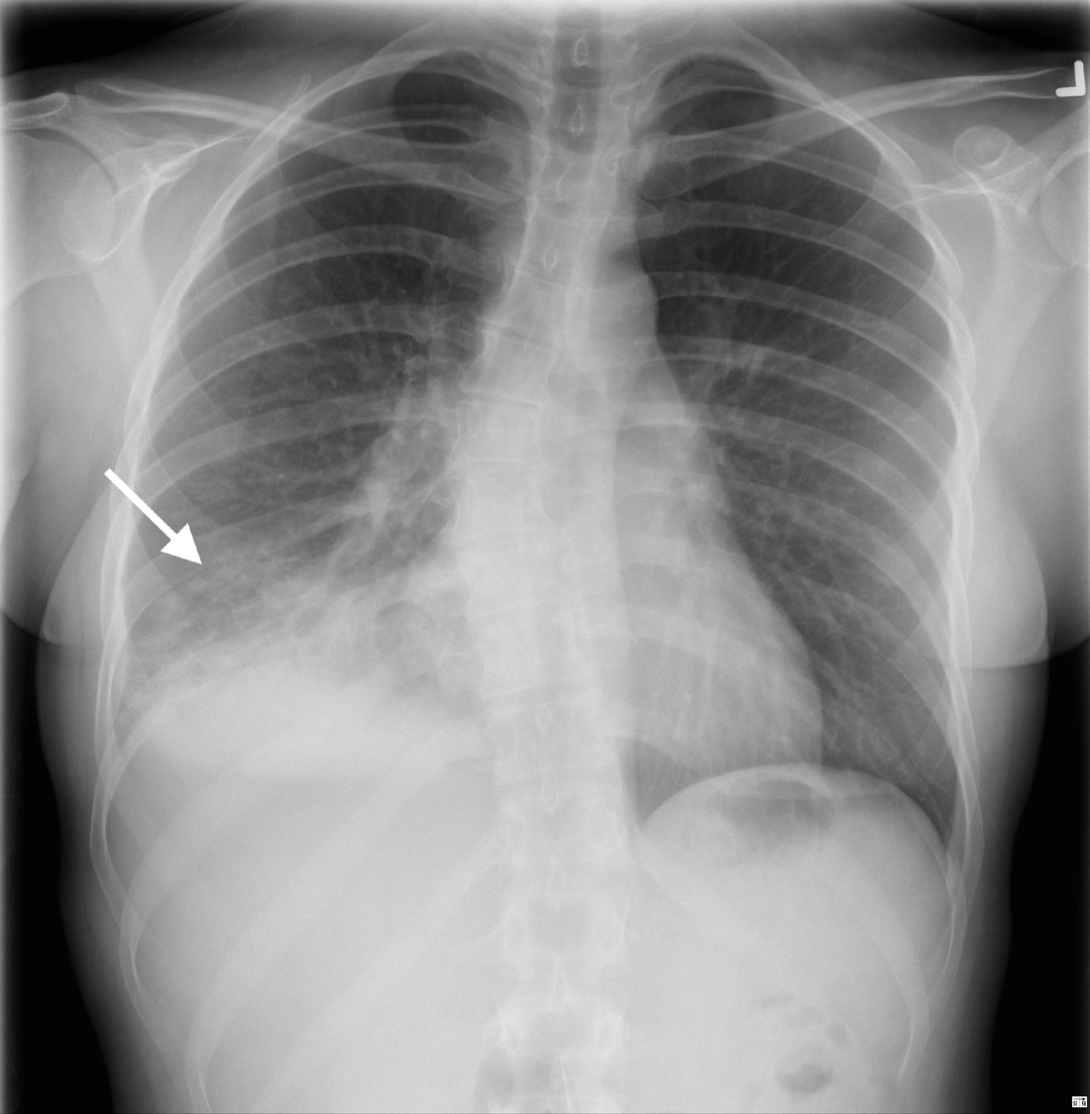
Anteroposterior portable chest radiograph: right lower lobe pneumonia.

While chest computed tomography (CT) is the gold standard diagnostic tool for CAP, its use is limited by both cost and radiation exposure.^2^ Unfortunately, chest radiography has poor sensitivity (43.5%) for the diagnosis of CAP when compared to CT (Figure 2).^3^ Lung ultrasound (LUS) has been shown to have superior sensitivity (80–95%), has no ionizing radiation, and is easy to perform at the bedside.^2^,^4^

On LUS, pneumonia has similar echogenicity to the liver with hyper-echoic foci, representing air bronchograms (Figure 1a). Consolidation allows transmission of ultrasound waves through the lung enabling visualization of the thoracic spine; this is known as the “spine sign.” In contrast, in a normal lung, air molecules scatter sound waves limiting their transmission and thus the spine is not visualized above the diaphragm (Figure 1b).

While it is underutilized, point-of-care LUS is a rapid, accessible, safe, and low-cost imaging tool for the diagnosis of pneumonia.^2^,^4^ LUS may be particularly useful in patients with high likelihood of a pneumonia but with a negative radiograph and for children to minimize radiation exposure. Practicing LUS in patients with a known infiltrate on radiograph may help providers increase their confidence and skills in the use of this growing diagnostic tool.

## Figures and Tables

**Figure 1a:**
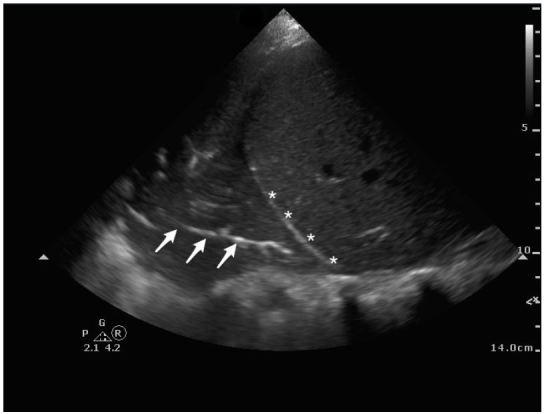
Ultrasound of lung with pneumonia: Linear, bright (hyper-echoic) foci represent air bronchograms (arrows) above the diaphragm (asterisks).

**Figure 1b:**
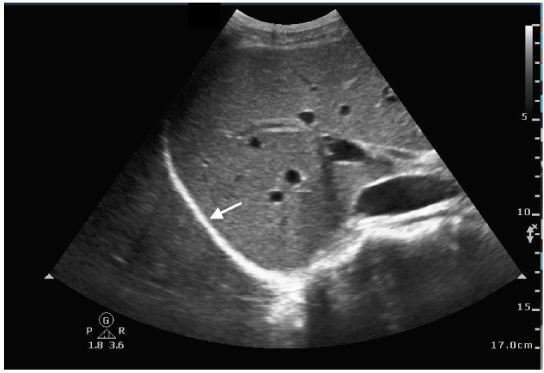
Ultrasound of normal lung: note the mirror image artifact: appearance of liver above and below the diaphragm (arrow) and the absence of a spine sign.

**Video:** Pneumonia on lung ultrasound.
